# Does Personality Shape COVID-19 Responses in Older Adults?

**DOI:** 10.1093/geroni/igab046.1210

**Published:** 2021-12-17

**Authors:** Damaris Aschwanden, Angelina Sutin, Amanda Sesker, Ji Hyun Lee, Martina Luchetti, Yannick Stephan, Antonio Terracciano

**Affiliations:** 1 Florida State University, Tallahassee, Florida, United States; 2 Florida State University College of Medicine, Tallahassee, Florida, United States; 3 University of Michigan, Ann Arbor, Michigan, United States; 4 University of Montpellier, Montpellier, Languedoc-Roussillon, France, 5, Florida State University, Florida, United States

## Abstract

Knowing how personality plays out in a pandemic can provide guidance to improve public health messaging. In a sample of 2066 participants (Mage = 51.42; 48.5% female), we examined whether personality is associated with concerns, precautions, preparations, and duration estimates of the COVID-19 pandemic. Personality traits were measured before the pandemic; responses were assessed in late March 2020. We investigated whether age moderates the trait-response associations because older adults are at higher risk for severe complications of COVID-19. Among the 65-96-year-olds, higher conscientiousness was associated with more preparations, higher openness was associated with greater concerns, and both higher openness and agreeableness were related to more preparations and longer duration estimates. This pattern has implications: If all older adults took COVID-19 seriously, individual differences in personality should not matter; however, our findings indicate that they do matter and could be considered in the development of personality-tailored communication to older adults.

